# Supplementary material for: High-quality sugar production by osgcs1 rice
Source: Commun Biol. 2020 Oct 27;3:617. doi: 10.1038/s42003-020-01329-x (PMC7592059; doi:10.1038/s42003-020-01329-x)
Supplement: Supplementary file 1 — Supplementary Information [file 42003_2020_1329_MOESM1_ESM.pdf]

High-quality sugar production by *osgcs1* rice

Yujiro Honma, Prakash Babu Adhikari, Keiko Kuwata, Tomoko Kagenishi, Ken Yokawa, Michitaka Notaguchi, Kenichi Kurotani, Erika Toda, Kanako Bessho-Uehara, Xiaoyan Liu, Shaowei Zhu, Xiaoyan Wu, Ryushiro D. Kasahara.

Supplementary information

1. Supplementary Figures.
Supplementary Figure 1. Maximum-likelihood phylogeny of eudicot GCS1 amino acids.
Supplementary Figure 2. Mutation sites and Alignment of AtGCS1 amino acids with the GCS1-orthologs of rice.
Supplementary Figure 3. Starch and sucrose metabolism related gene expression pattern in the *osgcs1* mutant.
Supplementary Figure legends.

2. Supplementary Table
Primers used in the study.

3. Supplementary Discussion.

4. Supplementary Data. (All data are deposited in an excel file.)
Raw Data. All RNA-Seq analysis data on the ovules of Nipponbare and osgcs1.
Supplementary Data 1. Data for early-response genes in cases of Nipponbare_0DAP = 0.
Supplementary Data 2. Data for early-response genes in cases of Nipponbare_0DAP > 0.
Supplementary Data 3. Data for the genes associated with cell expansion, cell division, and starch synthesis.
Supplementary Data 4. Data for the genes associated with starch metabolism.
Supplementary Data 5. Data for the genes associated with starch metabolism which was not highly upregulated in osgcs1 ovules compared to Nipponbare.

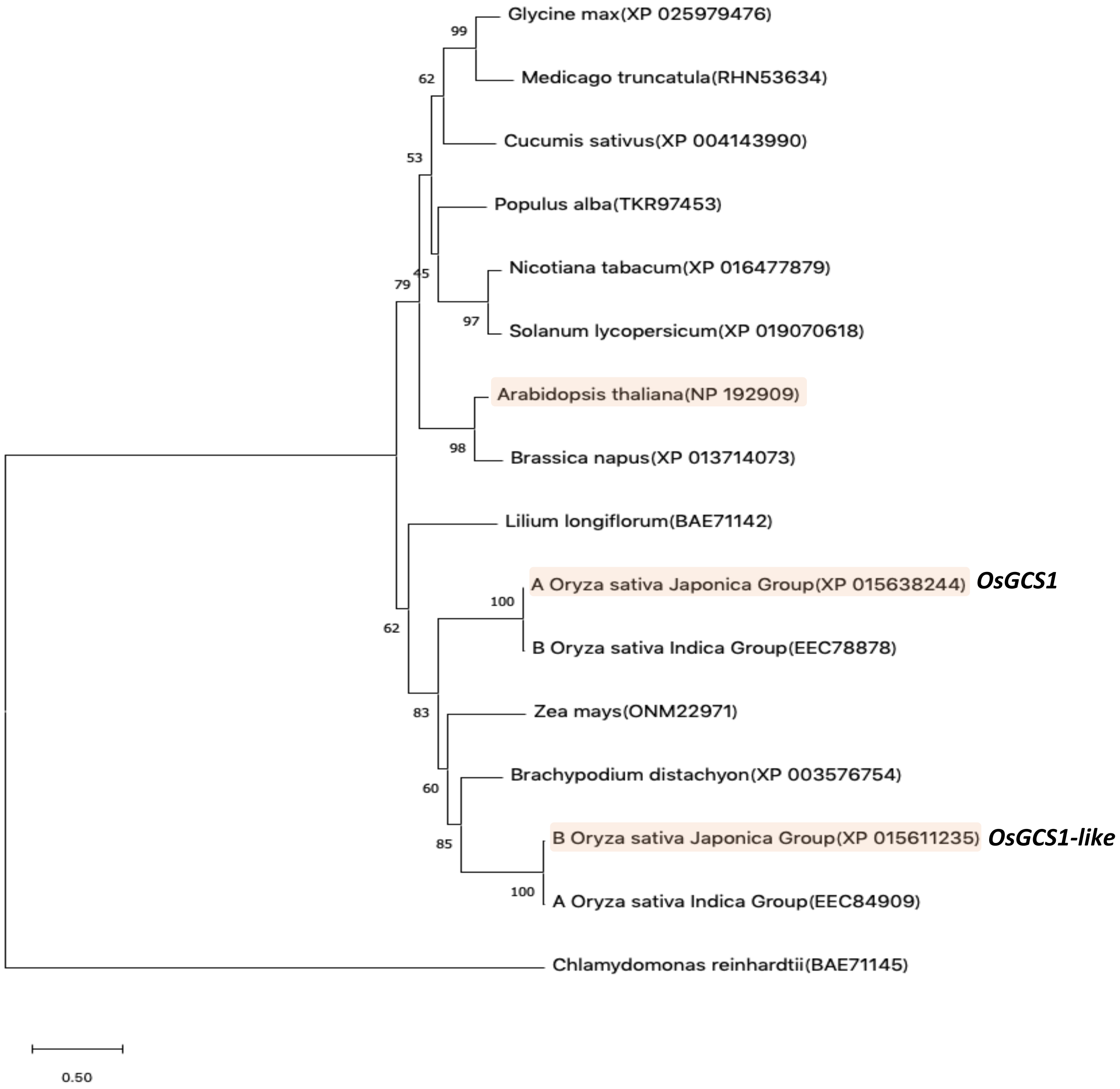
Glycine max(XP 025979476)
Medicago truncatula(RHN53634)
Cucumis sativus(XP 004143990)
Populus alba(TKR97453)
Nicotiana tabacum(XP 016477879)
Solanum lycopersicum(XP 019070618)
Arabidopsis thaliana(NP 192909)
Brassica napus(XP 013714073)
Lilium longiflorum(BAE71142)
A Oryza sativa Japonica Group(XP 015638244)
OsGCS1
B Oryza sativa Indica Group(EEC78878)
Zea mays(ONM22971)
Brachypodium distachyon(XP 003576754)
B Oryza sativa Japonica Group(XP 015611235)
OsGCS1-like
A Oryza sativa Indica Group(EEC84909)
Chlamydomonas reinhardtii(BAE71145)
99
62
53
45
79
97
98
100
62
83
60
85
100
0.50

a

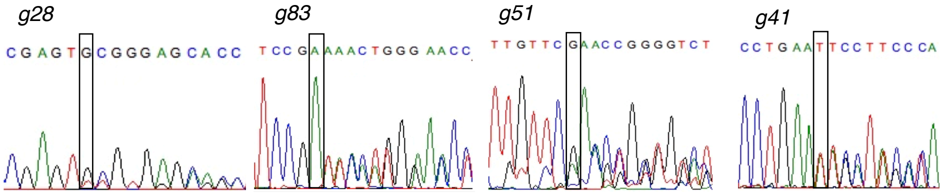

b

```
                                                         g28
OsGCS1       1   MPRRRGTPLP TILLLLAFVG GACGTEILSK SRLESCSHDS DAGGRLKCDR KLVVDLAVPS  60
OsGCS1-like  1   ---------- ---------- ---------- ---------- ---------- ----------  1
AtGCS1       1   MVNAILMACI LAGIFVGMFN EVDGIQILSK SKLEKCEKTS DSG-NLNCST KIVLNLAVPS  59

OsGCS1       61  GASGGEASLV ARVAGVEEEN DTPSATKSIR DPPVITVSKS ATYALYALTY LDRDVAYRPD  120
OsGCS1-like  1   ---------- ---------- ------MPIR DPLIITINKS EVYALYDLTY L-RDVAYKPE  33
AtGCS1       60  GSSGGEASIV AEIVEVEDNS S--SNMQTVR IPPVITVNKS AAYALYDLTY I-RDVPYKPQ  116

OsGCS1       121 EKYVKTHKCE PYAGAKVVGE CERLWDEKGN VIKQTEPICC PCGPHR-VQS KCGDIWSKLT  179
OsGCS1-like  34  EKFVKTRKCE PEAGANVVKS CERLRDEKGS IIEHTEPVCC PCGPHRRVPS SCGNILDKVA  93
AtGCS1       117 EYHVTTRKCE HDAGPDIVQI CERLRDEKGN VLEQTQPICC PCGPQRRMPS SCGDIFDKMI  176

OsGCS1       180 KGKANTAHCV RFPGDWFHVF GIGAWSLRFS IRVQVKKGSS VWDVVVGPEN KTVVSGDNFL  239
OsGCS1-like  94  KGKANTAHCL RFPDDWFHVF DIGRRSLWFS IRVQVKKGSS ESEVIVGPEN RTVVSEDSSL  153
AtGCS1       177 KGKANTAHCL RFPGDWFHVF GIGQRSLGFS VRVELKTGTR VSEVIIGPEN RTATANDNFL  236

OsGCS1       240 RVKVVGDYTG YTSIPSFEDN YLVTPRKGTG SSQPQDLGNE HSKWMILDRV RFTLDGLECD  299
OsGCS1-like  154 RVNLVGDFAG YTSLPSLENF YLVTPRKGVG GGQLEVLGDD FSRWMLLERV LFTLDGLECN  213
AtGCS1       237 KVNLIGDFGG YTSIPSFEDF YLVIPREAAE AGQPGSLGAN YSMWMLLERV RFTLDGLECN  296

                                       g83
OsGCS1       300 KIGVGYEAYR NQPNFCSAPY GSCLGNQLWN FWEYDKRRID NSQLPLYIVE GRFQRINQHP  359
OsGCS1-like  214 KIGVGYEAFR SQPNFCSSPL DSCLGDQLSK FWEIDKNRVN NSQPPQYVVL GKFERINQYP  273
AtGCS1       297 KIGVGYEAFN TQPNFCSSPY WSCLHNQLWN FRESDINRID RHQLPLYGLE GRFERINQHP  356

OsGCS1       360 NAGAHTFSVG VTEDLNTNLL IELMADDIEY VYQRSPAKII DIRVPTFEAL SQVGIANVTT  419
OsGCS1-like  274 NAGVHTFSVG IPEVLNTNLM IELSADDIEY VYQRSSGKII SINISSFEAL SQVGSARVKT  333
AtGCS1       357 NAGPHSFSIG VTETLNTNLM IELRADDIEY VFQRSPGKII NIAIPTFEAL TQFGVAAVII  416

                                                  g41
OsGCS1       420 KNIGKLESSY SLTFKCSSGI SPVEEQLYTM KPDEVIARSF ELRSTTDQAA MHQCEAILKA  479
OsGCS1-like  334 KNIGRLEASY SLTFDCLSGI NPVEEQYFIM KPDEKLIRTF DLRSSTDQAS NYTCQAILKA  393
AtGCS1       417 KNTGEVEASY SLTFDCSKGV AFVEEQFFII KPKAVTTRSF KLYPTKDQAA KYICTAILKD  476

OsGCS1       480 SDFSELDREG YRFSTAATVY NNGAQIGPTN DHKKG---GF WDSIKA---- LWRNLIDFLT  532
OsGCS1-like  394 SDFSELDRKE SQFSTTATVL NNGTQIGSSE NHTKGGIWGF FEAIKAWCAK MWHMLINFFT  453
AtGCS1       477 SQFSEVDRAE CQFSTTATVL DNGTQVTNPF QIPETQPKGF FDSIRILWTK IINGLVDFIT  536

                                                                        g51
OsGCS1       533 GRLCWTKCPR LFDFGCHIQY VCIGWI---- LLLLLIPAAV VFLWLLHQEG LFDPLYDWWG  588
OsGCS1-like  454 GTTCSTRCWS FLKFVIHG-- ---------- --LLLV---- AVLWLLHRKG LFDPLYYWWD  495
AtGCS1       537 GDTCRNKCSS FFDFSCHIQY VCLSWMVMFG LLLALFPITC LLLWLLHQKG LFDPCYDWWE  596

OsGCS1       588 --LEPDDDYR ARRRHQKGRH HRHHHDHRHR HGHSHGDHHH HYHGGHHQRR RHHHPPAWDV  646
OsGCS1-like  496 GVVGSEAQER ARRRHKRAHS HRHSHHH--- --DAHKRHKT ELAG----HR RHHVLHIHDD  546
AtGCS1       597 DHFDLDHHRR LLP-SRADVV NRHHHHH--- ---KHRHHHN HHRR---THQ RHKHHHGQDD  646

OsGCS1       647 EGHHHDRQQH SHEAGRNHHR GYGEVVAAGA APLRLDRASR PGQTEVDAVV EYRERRSRHE  706
OsGCS1-like  547 DDPVAAAAAA EHVILRRHGR HEAALGVQHR DGLKINKHRR HGGKAVALLP PGEIIVRDGG  606
AtGCS1       647 DVLQKMMLER DHSDSHYYHQ LH---RVHKD SKQKQRRRAK HG-----IVL PRDVHVER--  696

OsGCS1       707 RHGGHGHRDG HYSPSV 722
OsGCS1-like  607 GCGGVEHGDR RHHAWH 622
AtGCS1       696 ------QRKQ RLRES- 705
```

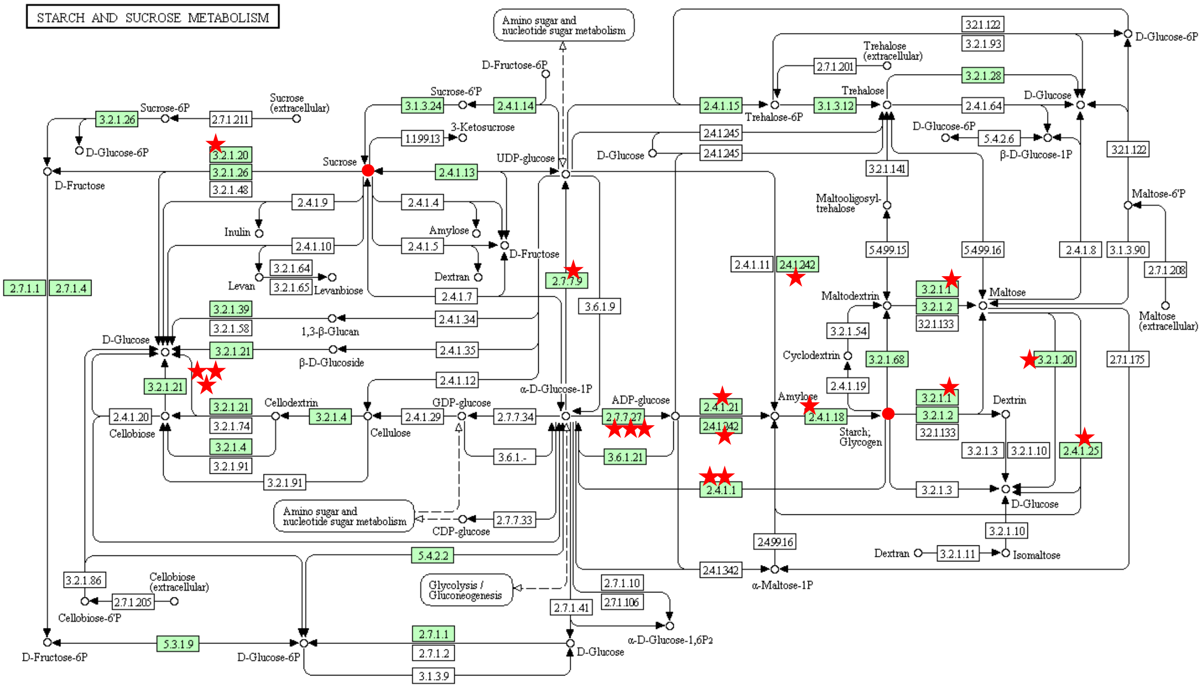
STARCH AND SUCROSE METABOLISM
Amino sugar and nucleotide sugar metabolism
D-Fructose-6P
Sucrose-6'P
2.4.1.14
3.1.3.24
Sucrose-6P
3.2.1.26
2.7.1.211
Sucrose (extracellular)
D-Glucose-6P
3.2.1.20
3.2.1.26
3.2.1.48
D-Fructose
Sucrose
1.1.99.13
3-Ketosucrose
2.4.1.13
UDP-glucose
2.4.1.9
2.4.1.4
Inulin
Amylose
2.4.1.10
2.4.1.5
D-Fructose
3.2.1.64
Levan
3.2.1.65
Levanbiose
Dextran
2.4.1.7
2.7.7.9
3.6.1.9
2.7.1.1
2.7.1.4
3.2.1.39
2.4.1.34
3.2.1.58
1,3-β-Glucan
D-Glucose
3.2.1.21
2.4.1.35
β-D-Glucoside
3.2.1.21
2.4.1.12
α-D-Glucose-1P
3.2.1.21
Cellodextrin
3.2.1.4
2.4.1.29
GDP-glucose
2.7.7.34
2.4.1.20
3.2.1.74
Cellulose
Cellobiose
3.2.1.4
3.6.1.-
3.2.1.91
3.2.1.91
Amino sugar and nucleotide sugar metabolism
CDP-glucose
2.7.7.33
5.4.2.2
3.2.1.86
Cellobiose (extracellular)
2.7.1.205
Cellobiose-6'P
Glycolysis / Gluconeogenesis
2.7.1.10
2.7.1.106
2.7.1.41
α-D-Glucose-1,6P2
D-Fructose-6P
5.3.1.9
D-Glucose-6P
2.7.1.1
2.7.1.2
D-Glucose
3.1.3.9
2.4.1.15
Trehalose-6P
D-Glucose
2.4.1.245
2.4.1.245
3.2.1.122
3.2.1.93
D-Glucose-6P
Trehalose (extracellular)
2.7.1.201
3.2.1.28
Trehalose
3.1.3.12
2.4.1.64
D-Glucose
D-Glucose-6P
5.4.2.6
β-D-Glucose-1P
3.2.1.141
3.2.1.122
Maltooligosyl-trehalose
Maltose-6'P
5.4.99.15
5.4.99.16
2.4.1.8
3.1.3.90
2.4.1.11
2.4.1.242
2.7.1.208
Maltodextrin
3.2.1.1
Maltose
3.2.1.2
Maltose (extracellular)
3.2.1.133
3.2.1.54
Cyclodextrin
3.2.1.68
3.2.1.20
2.7.1.175
2.4.1.19
ADP-glucose
2.4.1.21
Amylose
3.2.1.1
Dextrin
2.7.7.27
2.4.1.242
2.4.1.18
3.2.1.2
Starch; Glycogen
3.2.1.133
3.6.1.21
3.2.1.3
3.2.1.10
2.4.1.25
2.4.1.1
3.2.1.3
D-Glucose
3.2.1.10
2.4.99.16
Dextran
3.2.1.11
Isomaltose
2.4.1.342
α-Maltose-1P

Supplementary Figure 1. Maximum-likelihood phylogeny of eudicot GCS1 amino acids. The values near each node represent the strength of the associated sequences clustered together. The scale bar represents 0.2 estimated amino acid substitutions per residue. *AtGCS1*, *OsGCS1*, and *OsGCS1-like* sequences are marked with bold letters. BAE71145 (derived from *Chlamydomnas reinhardtii*) was used as an outgroup (*bootstrap = 1000 replications*).

Supplementary Figure 2. Mutation sites and Alignment of AtGCS1 amino acids with the GCS1-orthologs of rice. (**a**) Nucleotide sequences of CRISPR/Cas9-derived mutation in respective mutants. Black rectangles indicate deletion mutation starting sites. (**b**) Peptide N-terminal signal sequence (SS) is highlighted in gray, the HAP2/GCS1 domain is boxed in yellow; transmembrane domain (TD) is boxed in red, and C-terminal histidine rich domains are boxed in green (H1, H2, H3, and H3.1 in order from N-terminus to C-terminus). Apparently, OsGCS1-like peptide lacks almost all of the TD while AtGCS1 lacks almost all of the H3.1 region.

Supplementary Figure 3. Starch and sucrose metabolism related gene expression pattern in the *osgcs1* mutant. The entire metabolic pathway for starch and sucrose synthesis was drawn by reference to the KEGG PATHWAY database (https://www.genome.jp/kegg/pathway.html). The EC numbers in boxes indicate the classes of enzymes related to the corresponding catalytic steps. The catalytic enzymes highlighted in green are those identified in rice. Down-regulated genes in *osgcs1* ovules compared to WT ovules are marked as red stars (the number of stars in each catalytic step indicates the number of down-regulated genes in *osgcs1* ovules).

Supplementary Table 1: Primers used in the study

SN	Primer ID	Primer sequence
1	*g28*-CRISPR.fwd	5′-GTTGCGAGAGCTGCTCCCACGACTCGG-3′
2	*g28*-CRISPR.rev	5′-AAACCCGAGTCGTGGGAGCAGCTCTCG-3′
3	*g83*-CRISPR.fwd	5′-AAACGTTGTCCTCAAATGATGGGA-3′
4	*g83*-CRISPR.rev	5′-CTTGTCCCATCATTTGAGGACAAC-3′
5	*g51*-CRISPR.fwd	5′-AAACTCGTACAGCGGGTCGAACAA-3′
6	*g51*-CRISPR.rev	5′-CTTGTTGTTCGACCCGCTGTACGA-3′
7	*g41*-CRISPR.fwd	5′-GTTGGAGTTGGCTATGAAGCTTTCAGG-3′
8	*g41*-CRISPR.rev	5′-AAACCCTGAAAGCTTCATAGCCAACTC-3′
9	*OsGCS1_qRT.fwd*	5′-CCATGAAACCTGATGAAGTG-3′
10	*OsGCS1_qRT.rev*	5′-CTAAAGTCTGATGCCTTCAG-3′
11	*OsGCS1-like_qRT.fwd*	5′-CGTCAACAGACCAAGCATCG-3′
12	*OsGCS1-like_qRT.rev*	5′-GAACTGTAGCTGTGGTTGAG-3′
13	*OsAct_qRT.fwd*	5′-CTTGACCCTCAAGTACCCCA-3′
14	*OsAct_qRT.rev*	5′-GGCCACACGGAGCTCGTTGT-3′

Supplementary Discussion:

Further analyses for the transcriptome data.

The globular stage in rice is reported to last only until 3DAP. Among various other genes, *CYP78A13* was relatively highly up-regulated in the *osgcs1* ovules. The gene reportedly plays a role in embryo size control and endosperm size facilitation in rice [1]. Its upregulation could be one of the reasons why *osgcs1* ovules grow and develop to a size comparable with that of Nipponbare rice grains despite lacking endosperm structure within.

While the expression of most of the starch biosynthesis-related differently expressed genes (DEGs) dropped from 1DAP to 3DAP in *osgcs1* ovules, their expression in WT ovules was markedly enhanced (Fig. 2 and Extended Data Fig. 3). Among these DEGs were the genes putatively involved in starch granule formation, development, and maturation, (*Os03t0758100*, a member of glycosyl transferase family 35), amylose and amylopectin biosynthesis (*Os07t0412100*, a member of glycosyltransferase family 1; and *Os09t0469400*, a member of glycosyl hydrolase family 13) (Fig. 2 and Extended Data Fig. 3). Amylose and amylopectin are the two components of starch. Significantly lower expression of the aforementioned genes (and of other genes involved in starch biosynthesis) in *osgcs1* ovules at 3DAP (and most likely at later stages as well) strongly suggests that *osgcs1* ovules produce negligible amounts of starch and accumulate high concentrations of sucrose. Interestingly, an earlier study[2] showed similar expression levels for glycosyltransferase and glycosyl hydrolase family member genes in *Arabidopsis* but, as the POEM ovules were aborted early in their development, their status regarding sugar content was not taken into account then. However, it is important to note that the final sink compound in normal rice grains is starch while that in *Arabidopsis* seed is oil. Additionally, the Nipponbare and *osgcs1* rice ovules showed different expression profiles for the DEGs involved in auxin biosynthesis and/or response, indicating the involvement of several auxin-related genes during normal rice seed formation. In contrast to the earlier study on *Arabidopsis*, though, we did not find any significant difference in the expression profiles of genes related to cyclin, and expansin (Fig. 2).

References:

1. Nagasawa N. *et al. GIANT EMBRYO* encodes CYP78A13, required for proper size balance between embryo and endosperm in rice. *The Plant Journal* **75**, 592-605 (2013).
2. Kasahara, R. D. *et al.* Pollen tube contents initiate ovule enlargement and enhance seed coat development without fertilization. *Science Advances* **2**, e1600554 (2016).
