# Supplementary material for: High-quality sugar production by osgcs1 rice
Source: Commun Biol. 2020 Oct 27;3:617. doi: 10.1038/s42003-020-01329-x (PMC7592059; doi:10.1038/s42003-020-01329-x)
Supplement: Supplementary file 2 — Description of Additional Supplementary Files [file 42003_2020_1329_MOESM2_ESM.pdf]

Description of Additional Supplementary Files

File Name: Supplementary Data 1-5
Description:
Raw Data. All RNA-Seq analysis data on the ovules of Nipponbare and osgcs1.
Sup. Data 1. Data for early-response genes in cases of Nipponbare_0DAP = 0.
Sup. Data 2. Data for early-response genes in cases of Nipponbare_0DAP > 0.
Sup. Data 3. Data for the genes associated with cell expansion, cell division, and starch synthesis.
Sup. Data 4. Data for the genes associated with starch metabolism.
Sup. Data 5. Data for the genes associated with starch metabolism which was not highly upregulated in osgcs1 ovules compared to Nipponbare.
